# Analyzing Question Characteristics Influencing ChatGPT’s Performance in 3000 USMLE®-Style Questions

**DOI:** 10.1007/s40670-024-02176-9

**Published:** 2024-09-28

**Authors:** Michael Alfertshofer, Samuel Knoedler, Cosima C. Hoch, Sebastian Cotofana, Adriana C. Panayi, Martin Kauke-Navarro, Stefan G. Tullius, Dennis P. Orgill, William G. Austen, Bohdan Pomahac, Leonard Knoedler

**Affiliations:** 1 Department of Oral and Maxillofacial Surgery, Ludwig-Maximilians-University Munich, Munich, Germany; 2 Division of Plastic Surgery, Brigham and Women’s Hospital, Harvard Medical School, Boston, MA USA; 3 Division of Plastic Surgery, Yale New Haven Hospital, Yale School of Medicine, New Haven, CT USA; 4 Department of Otolaryngology, Head and Neck Surgery, Technical University of Munich, Munich, Germany; 5 Department of Dermatology, Erasmus Hospital, Rotterdam, The Netherlands; 6 Centre for Cutaneous Research, Blizard Institute, Queen Mary University of London, London, UK; 7 Department of Hand, Plastic and Reconstructive Surgery, Microsurgery, Burn Trauma Center, BG Trauma Center Ludwigshafen, University of Heidelberg, Ludwigshafen, Germany; 8 Division of Transplant Surgery, Brigham and Women’s Hospital, Harvard Medical School, Boston, MA USA; 9 Division of Plastic and Reconstructive Surgery, Massachusetts General Hospital, Harvard Medical School, Boston, MA USA; 10 Department of Plastic, Hand and Reconstructive Surgery, University Hospital Regensburg, Regensburg, Germany; 11 Department of Plastic Surgery and Hand Surgery, Klinikum Rechts der Isar, Technical University of Munich, Munich, Germany

**Keywords:** Medical education, Artificial intelligence, ChatGPT, USMLE, Quiz

## Abstract

**Background:**

The potential of artificial intelligence (AI) and large language models like ChatGPT in medical applications is promising, yet its performance requires comprehensive evaluation. This study assessed ChatGPT’s capabilities in answering USMLE® Step 2CK questions, analyzing its performance across medical specialties, question types, and difficulty levels in a large-scale question test set to assist question writers in developing AI-resistant exam questions and provide medical students with a realistic understanding of how AI can enhance their active learning.

**Materials and Methods:**

A total of *n*=3302 USMLE® Step 2CK practice questions were extracted from the AMBOSS© study platform, excluding 302 image-based questions, leaving 3000 text-based questions for analysis. Questions were manually entered into ChatGPT and its accuracy and performance across various categories and difficulties were evaluated.

**Results:**

ChatGPT answered 57.7% of all questions correctly. Highest performance scores were found in the category “Male Reproductive System” (71.7%) while the lowest were found in the category “Immune System” (46.3%). Lower performance was noted in table-based questions, and a negative correlation was found between question difficulty and performance (*r*_s_=−0.285, *p *<0.001). Longer questions tended to be answered incorrectly more often (*r*_s_=−0.076, *p *<0.001), with a significant difference in length of correctly versus incorrectly answered questions.

**Conclusion:**

ChatGPT demonstrated proficiency close to the passing threshold for USMLE® Step 2CK. Performance varied by category, question type, and difficulty. These findings aid medical educators make their exams more AI-proof and inform the integration of AI tools like ChatGPT into teaching strategies. For students, understanding the model’s limitations and capabilities ensures it is used as an auxiliary resource to foster active learning rather than abusing it as a study replacement. This study highlights the need for further refinement and improvement in AI models for medical education and decision-making.

## Introduction

Artificial intelligence (AI) combines machine learning mechanisms and deep learning strategies with automated processing of tasks that traditionally required human intelligence. Machine learning involves developing algorithms and statistical models to extract patterns from large databases, enabling automated, analytic predictions, and decision-making [1, 2]. Deep learning techniques employ artificial neural networks with multiple layers to address complex problems deployed for a plethora of medical scenarios including automating grading tasks, evaluating clinical patient data, and simulating surgical outcomes before surgery [3], [4], [5].

Recently, ChatGPT has emerged as a revolutionary chatbot that uses a large language model and deep learning to generate analytic human-like responses to both medical and non-medical questions. Kung et al. reported that ChatGPT achieved performance levels at, or near, the passing threshold of approximately 60% for all three United States Medical Licensing Exam (USMLE®) steps without any specialized training [6]. These performance scores were further substantiated by larger-scale studies for Step 1, Step 3, and basic science as well as shelf examination questions, thereby effectively showcasing ChatGPT’s potential in medical test taking [7–9]. The USMLE® is a standardized test required for medical licensure in the USA, consisting of three steps with the first two exams commonly taken by second- and fourth-year medical students, respectively; Step 3 is typically taken by physicians with at least 6 months of postgraduate medical experience. Notably, USMLE® scores have been shown to influence the physician’s future career and impact the residency matching process significantly [10–12]. With the recent transition of USMLE® Step 1 to a pass/fail system, the residency matching process is thought to now place greater emphasis on USMLE® Step 2 CK scores as the pivotal objective parameter [13, 14].

Hence, a multi-faceted and comprehensive insight into the performance of ChatGPT on medical examinations is warranted. Specifically, there is a knowledge gap investigating the capabilities of ChatGPT on USMLE**®** Step 2CK questions in a large-scale study. Assessing ChatGPT’s performance on USMLE® Step 2CK preparatory questions is essential for evaluating its ability to handle clinically relevant, real-world scenarios integral to medical education. These insights can help educators understand how AI tools might augment traditional training and guide medical students in using such tools to support active learning rather than replace genuine knowledge acquisition [15, 16]. For educators, understanding the strengths and limitations of AI is key for educators to create AI-resistant practice questions that accurately assess students’ knowledge, ensuring robust and meaningful evaluations. Hence, data of our study may provide another puzzle piece in understanding and leveraging the use of AI and ChatGPT to advance medical education, guide clinical decision-making, and integrate cutting-edge technologies into healthcare.

## Methods

### Question Bank Access and ChatGPT Data Entry

From May 22, 2023, to May 29, 2023, the online question bank AMBOSS© (New York, NY, USA) was accessed and 3302 USMLE® Step 2CK practice questions were extracted. All sample test questions were screened independently by four examiners (M.A.; S.K.; C.C.H.; L.K.), and image-based questions were removed, as ChatGPT 3.5 lacked the capability to analyze images at the time of the study. Following the removal of 302 image-based questions, 3000 test questions were classified according to the question type and specialty based on the platform’s proprietary categorization. To further subcategorize the test questions, the question format was identified, with the following five question types: standard, table-based, communications-based, follow-up, and patient vignette. All questions included in this study were designed as multiple-choice single-answer style questions. Test question difficulty was defined as the number of AMBOSS© hammers (one to five hammers; one hammer = the easiest 20% of all questions; five hammers = the most challenging 5% of all questions). Further, test question difficulty was assessed based on the human AMBOSS© peer group user performance (i.e., all AMBOSS© users taking the respective question) with the following five categories: 100–80% vs. 79–60% vs. 59–40% vs. 39–20% vs. 19–0.0%. Official permission for the use of these practice questions was obtained from AMBOSS© (Amboss GmbH, Berlin, Germany) prior to the initiation of the study. Two examiners (M.A.; L.K.) randomly spot-checked the inputs to ensure that none of the answers was indexed on Google prior to May 29, 2023, representing the last date accessible to the ChatGPT training dataset. One examiner (M.A.) then manually entered the test questions into ChatGPT version 3.5 (OpenAI, San Francisco, CA, USA). For this, an exact replication of the original question text and answer choices was copied and pasted in text format. For table-based questions, tables were entered in a standardized fashion as pasting them would have distorted their format. Further, the authors refrained from adding any instructing supplementary information and prompts to ChatGPT, thereby reducing the potential for systematic errors and methodological bias. For each question, a fresh chat session was initiated in ChatGPT to minimize the influence of memory retention bias.

ChatGPT was chosen for this study due to its status as one of the most widely used and recognized AI platforms, with more than 180 million users in total [17]. Its popularity and versatility across various applications make it a relevant tool to explore for educational purposes, especially in the context of medical education. Given its wide accessibility and the growing reliance on AI technologies, it was crucial to investigate how ChatGPT could be leveraged to benefit medical students and educators, enhancing both learning and assessment environments. The authors chose to employ ChatGPT 3.5 for a multitude of reasons: its wider user base makes it more representative of the general population, restricted access of ChatGPT 4, limitations on ChatGPT 4’s search queries cooldown (i.e., 40 search queries every 3 h) affecting the overall study duration and feasibility, and the opportunity to establish a foundational benchmark for future comparative studies.

An example standard test question includes:

*“A 10-year-old boy is brought for a follow-up examination 2 days after he was seen in the emergency department because of hives, hoarseness, and light-headedness. His symptoms began 15 minutes after he was stung by a bee and lasted approximately 60 minutes; they resolved before he was treated. He has been stung by bees three times over the past year, and each reaction has been more severe. Examination shows no abnormalities. Which of the following is the most appropriate recommendation to prevent future morbidity and mortality from this condition?*

(A) *Avoid areas known to have bees*
(B) *Avoid wearing colorful clothing outside*
(C) *Carrying diphenhydramine tablets*
(D) *Carrying self-injectable epinephrine*
(E) *Seek immediate medical attention following any future sting*”

**Fig. 1:**
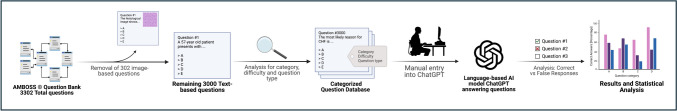
Comprehensive depiction of the study’s workflow

ChatGPT’s responses were recorded and the answer option was afterwards selected in the online platform. Subsequently, data regarding the accuracy of the responses, along with the percentage of the AMBOSS© user peer group for the correct and eventually incorrect answer (if ChatGPT chose the incorrect answer), were collected in a separate data spreadsheet. A comprehensive depiction of the entire study’s workflow can be found in Figure 1.

### Statistical Analysis

Pearson’s chi-square test was employed to calculate differences between question style and categories. Bivariate correlation analysis between ChatGPT performance and test question length and difficulty was performed using Spearman’s correlation coefficient (*r*_s_). Statistical analysis was conducted with IBM SPSS Statistics 25 (IBM, Armonk, NY), and a two-tailed *p*-value of ≤ 0.05 was deemed to guide statistical significance.

## Results

### General Test Question Characteristics and Performance Statistics

**Fig. 2:**
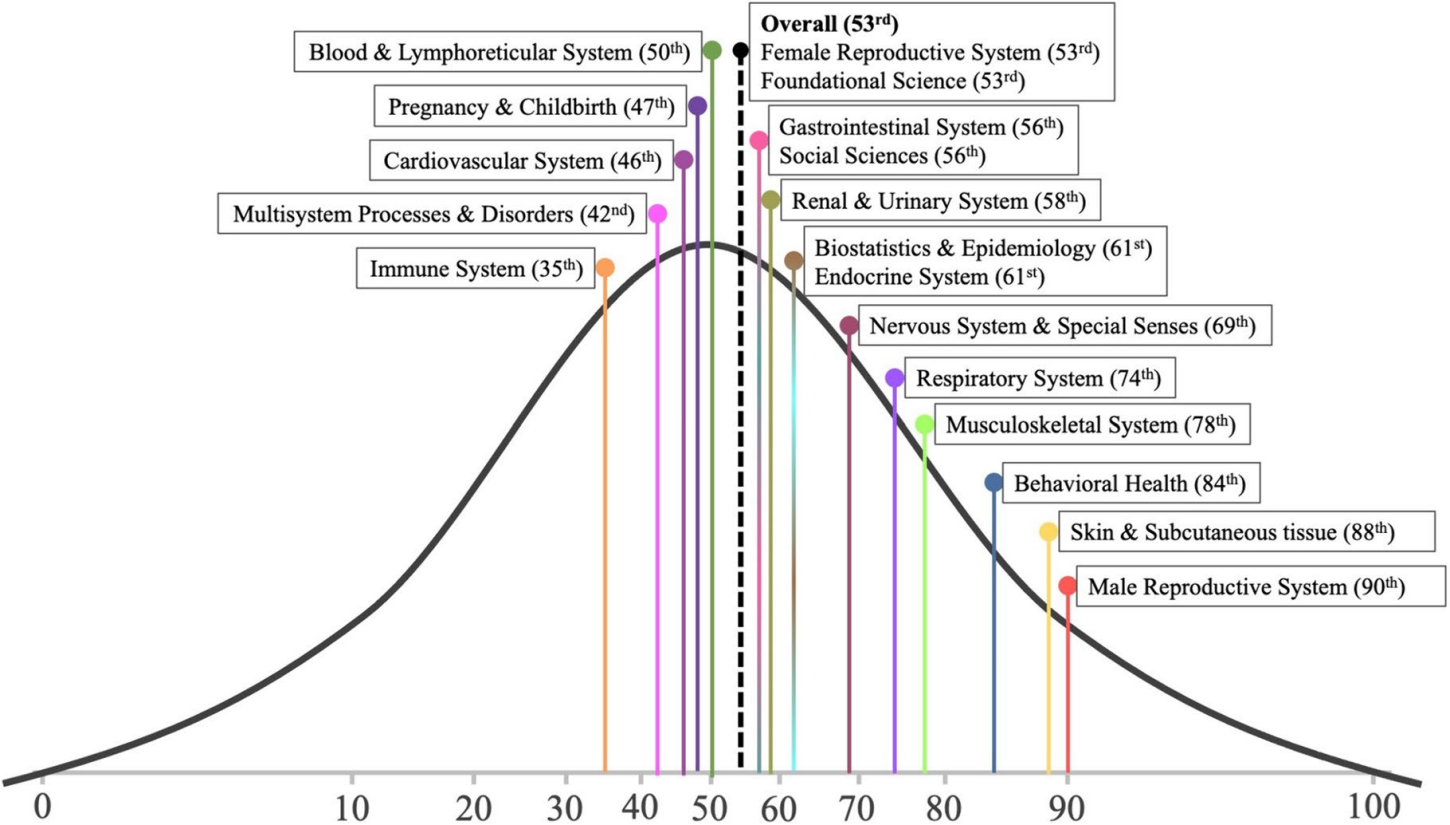
ChatGPT’s percentile performance related to the AMBOSS © user peer group sorted by specialties

**Fig. 3:**
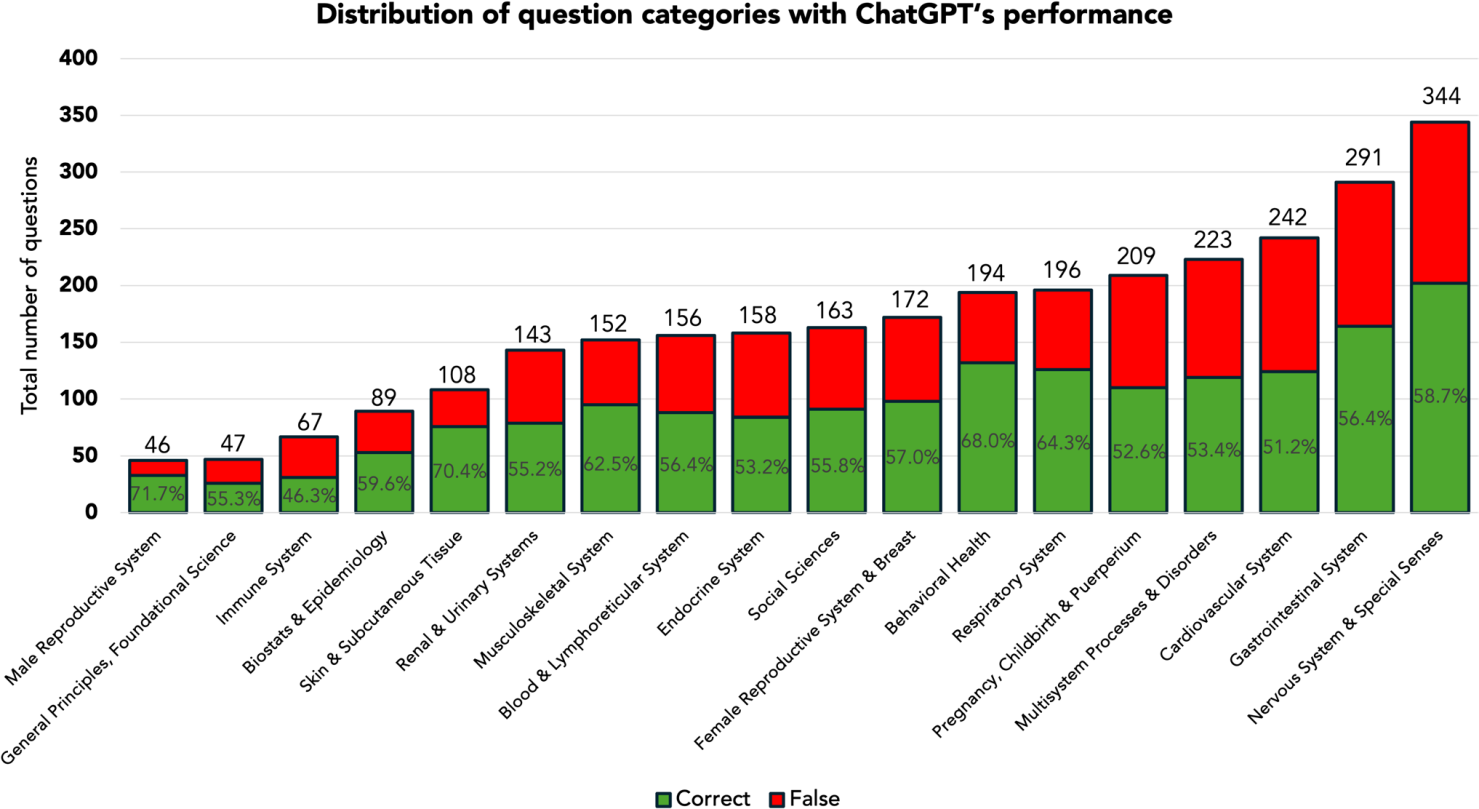
Distribution of test question specialties with absolute numbers and ChatGPT’s respective performance

**Fig. 4:**
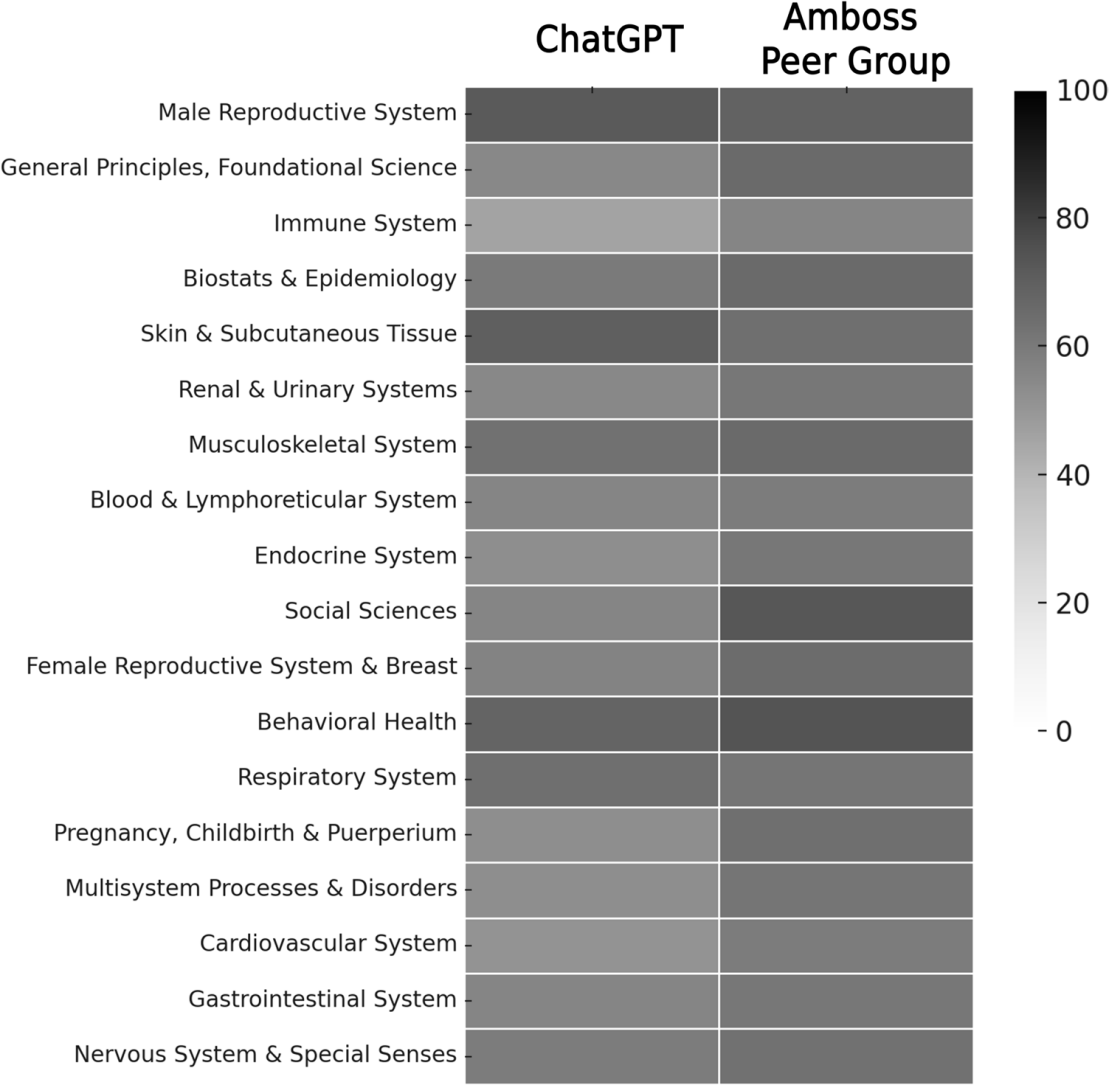
Heatmap comparing the performance of ChatGPT and the AMBOSS user peer group sorted by specialty

**Fig. 5:**
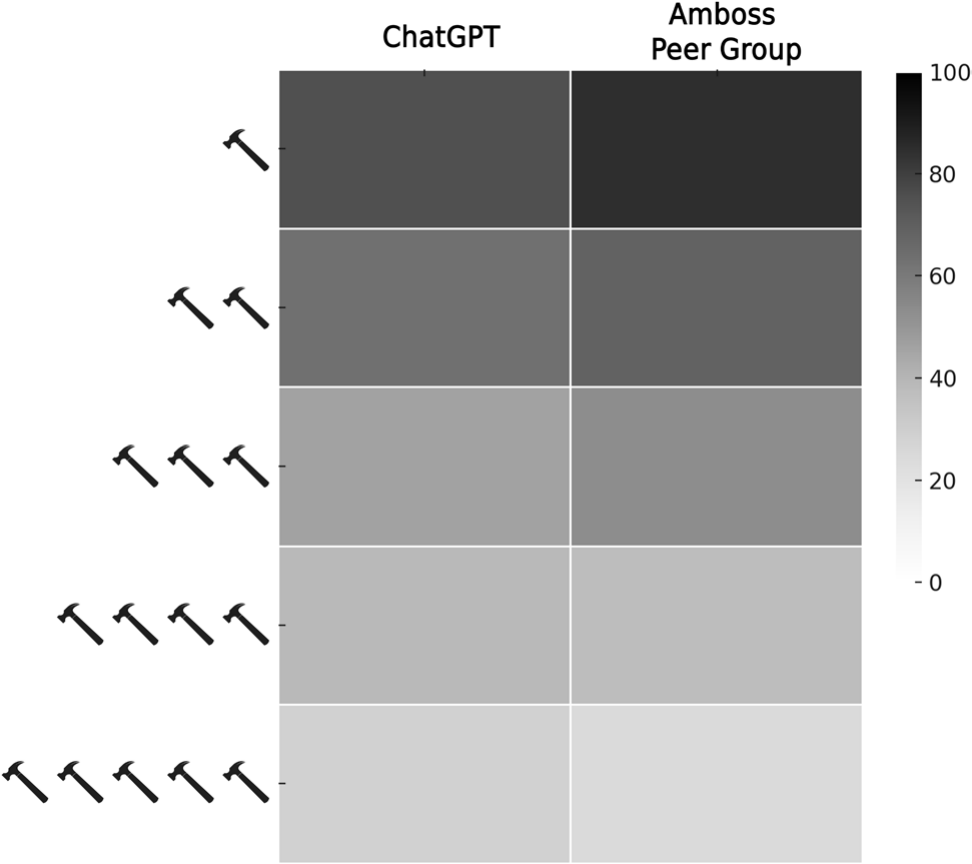
Heatmap comparing the performance of ChatGPT and the AMBOSS user peer group sorted by test question difficulty

ChatGPT overall accuracy for USMLE® Steps 2CK was 57.7% (1731/3000). ChatGPT’s specialty-specific overall and percentile performance related to the AMBOSS © user peer group are illustrated in Figures 2 and 3, respectively. A performance comparison between the AMBOSS© user peer group and ChatGPT sorted by specialty and test question difficulty is illustrated in Figures 4 and 5, respectively.

### Test Question Type and ChatGPT Performance

**Fig. 6:**
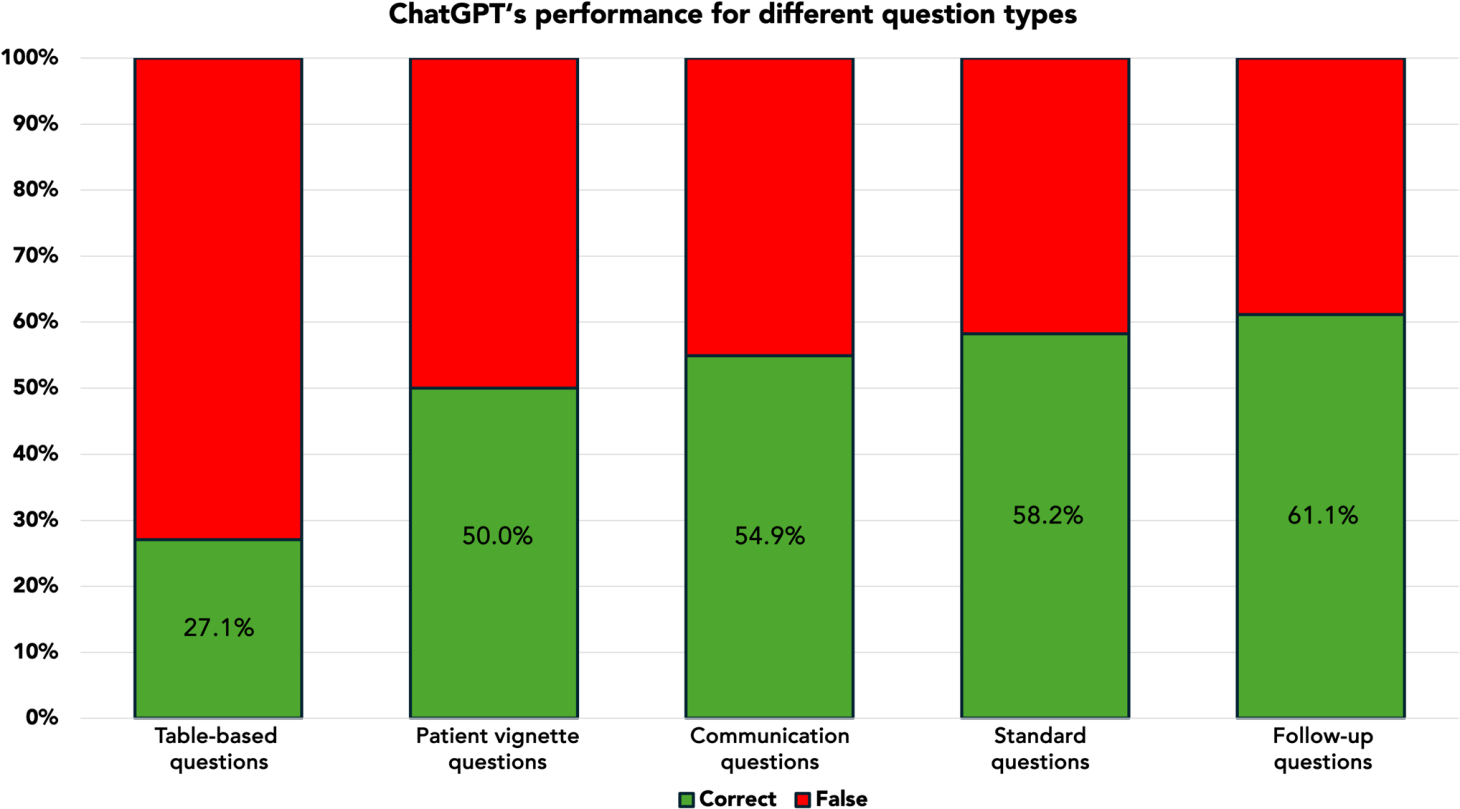
ChatGPT’s performance sorted by question type

The set of test questions consisted of 48 (1.6%) table-based, 72 (2.4%) follow-up, 51 (1.7%) communications, and 12 (0.4%) patient vignette questions, with the remaining questions falling into the standard questions category. Table questions yielded significantly lower ChatGPT performance scores when compared to standard questions (27.1% vs. 58.2%; *p *<0.001), while the remaining test question types did not show significantly different ChatGPT scores compared to standard questions (*p*>0.565). ChatGPT’s performance per question type is summarized in Figure 6.

### Test Question Difficulty and ChatGPT Performance

**Fig. 7:**
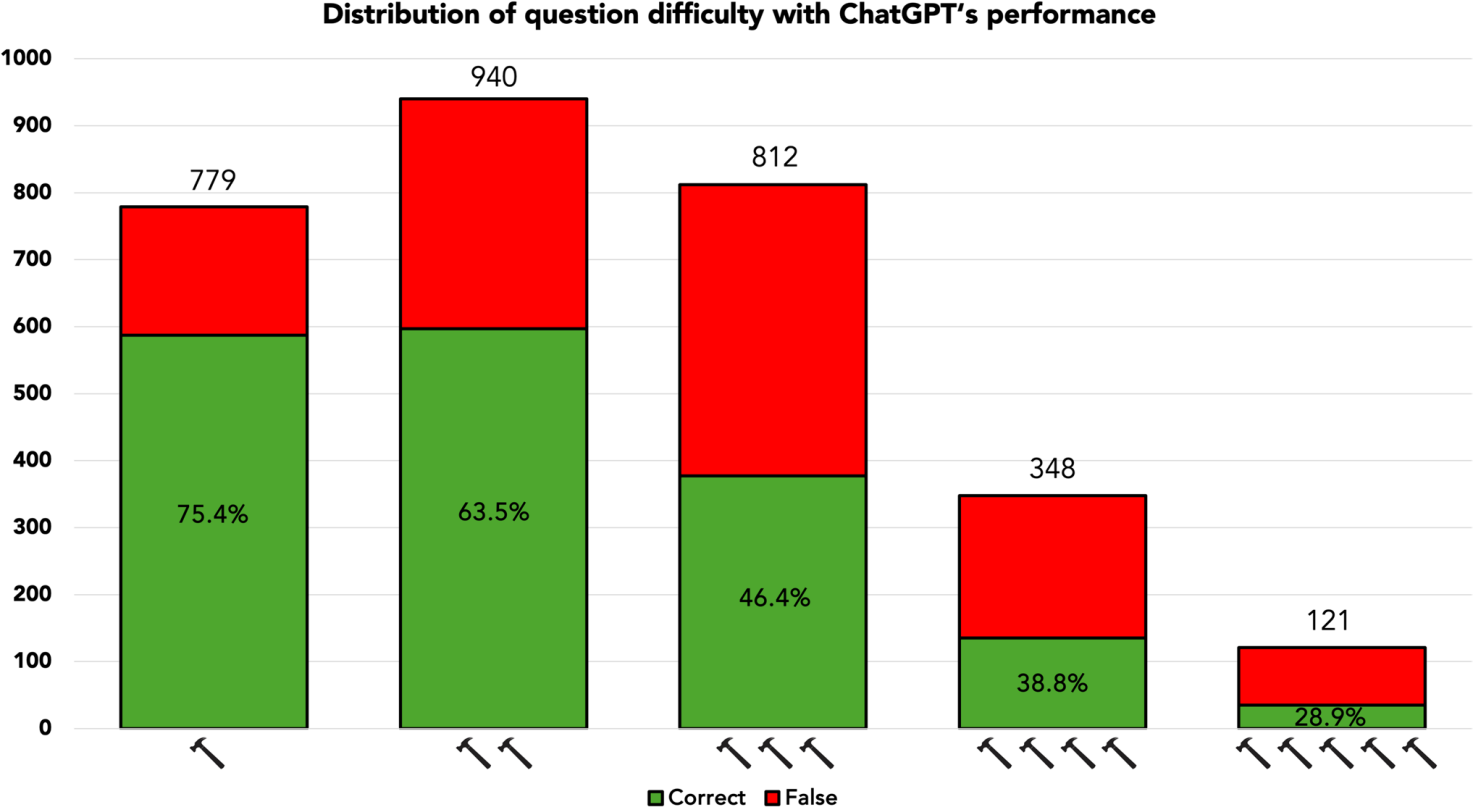
Distribution of test question difficulty with absolute numbers and ChatGPT’s respective performance

Test question difficulty defined by AMBOSS© hammer categorization and ChatGPT performance correlated significantly (*r*_s_=−0.285; *p *<0.001). Similarly, USMLE® Step 2CK question difficulty defined by human AMBOSS© peer group users and ChatGPT performance correlated significantly (*r*_s_=−0.290; *p *<0.001). ChatGPT’s performance sorted by test question difficulty is shown in Figure 7.

### Test Question Length and ChatGPT Performance

Test question length and ChatGPT performance correlated significantly (*r*_s_=−0.076; *p *<0.001) with a mean character number of 1304 ± 359 for correct answers, compared to 1346 ± 325 for incorrectly answered questions (*p *=0.001).

### Peer Group Responses Versus ChatGPT Performance Scores

When answering USMLE® Step 2 CK questions correctly, ChatGPT chose the answer that had been selected by 68.2 ± 16.7% of human AMBOSS© peer group users. At the same time, ChatGPT chose the answer that had been selected by 16.1 ± 12.3% of human AMBOSS© peer group users, when answering test questions incorrectly. In this scenario, 57.2 ± 17.7% of human AMBOSS© peer group users selected the correct answer (*p *<0.001).

### Multivariable Analysis of ChatGPT Performance Factors

Multivariable analysis confirmed that question specialty (*p *=0.001) and level of question difficulty defined by human AMBOSS© peer group users (*p *=0.009) impacted ChatGPT performance scores significantly.

## Discussion

In the present study, we conducted an in-depth investigation to assess the accuracy of ChatGPT in answering USMLE® Step 2CK practice questions sourced from the AMBOSS© question bank. The questions in the AMBOSS question bank are developed and continuously updated by a team of experienced US physicians and medical educators. The validation of these questions involves expert review, field testing with medical students, and an analysis of performance metrics like correct answer percentages and question difficulty [18]. The effectiveness of AMBOSS in approximating actual USMLE questions is evidenced by a study of nearly 2000 medical students from 159 universities. This study showed that students who primarily used AMBOSS for Step 2 CK preparation scored higher than those using other question banks, even surpassing the “gold standard” Uworld (AMBOSS, +10.4 points vs. Uworld 8.3 points), underscoring AMBOSS’s alignment with USMLE standards and efficacy in exam preparation [19]. The objective of this study was to rigorously evaluate ChatGPT’s performance in addressing complex medical scenarios. We conducted a thorough analysis to identify and characterize the specific question attributes that influence its performance.

ChatGPT achieved an overall performance rate of 57.7% (1731 out of 3000 questions), which fell slightly below the passing threshold of approximately 60%. These results are consistent with prior studies conducted by Kung et al. and Gilson et al., where ChatGPT scored 59.1% (60 out of 102 questions) and 57.8% (59 out of 102 questions) respectively for freely accessible USMLE® Step 2CK practice questions [6, 20]. However, our test question set included a thirtyfold number of test questions provided by one of the most renowned USMLE® test preparation platforms. Thus, our large-scale findings corroborate ChatGPT proficiency in approaching the passing threshold. Nonetheless, neither in the studies conducted by Kung et al. and Gilson et al. nor in the study presented herein was ChatGPT able to pass USMLE® Step 2CK. To gain a deeper understanding of ChatGPT performance, we compared it to the human AMBOSS© peer group user performance. This comparative analysis positioned ChatGPT as an average test taker, ranking at the 53^rd^ percentile. These findings suggest that approximately half of the test takers would have outperformed ChatGPT, while the remaining half would have achieved lower scores.

When expanding our analysis, a multitude of interesting and clinically relevant findings were revealed. Specifically, ChatGPT exhibited significantly lower proficiency in handling table-based questions compared to standard questions, with a performance rate of 27.1% versus 58.2%, respectively, with *p* < 0.001, thereby barely scoring higher than randomly guessing the answer. Table-based questions require the selection of appropriate values or relationships for various parameters, serving as a litmus test for one’s comprehensive understanding of physiological relationships within the human body. For instance, a question might pertain to cardiogenic shock and inquire about the anticipated alterations (increased vs. physiological vs. decreased) in systemic vascular resistance, left atrial pressure, and systolic blood pressure. ChatGPT relatively lower performance in this question type can be attributed to its limited grasp of the integrative aspect required for such questions. Successfully answering table-based questions often relies on the inclusion and exclusion of answer choices based on individual patient parameters, necessitating a careful evaluation of anticipated changes for each variable in the question stem to arrive at the correct answer. Such questions demand not only recognize patterns but also understand how these parameters interact with each other in specific pathophysiological contexts, which poses a unique challenge for ChatGPT’s capabilities. It appears that ChatGPT is still in the process of acquiring the necessary skills to effectively tackle these types of questions [21].

Another intriguing aspect emerged when we observed a consistent decline in ChatGPT performance with increasing question difficulty, mirroring the performance pattern observed in the AMBOSS© user peer group. Previous studies have reported a statistically significant relationship between ChatGPT test-taking performance and question difficulty [7, 8, 20]. Despite being an artificial intelligence model designed to learn from human responses, ChatGPT’s knowledge foundation inherently reflects the common errors made by human test takers with its reliance on pattern recognition from previously scraped data potentially explaining its difficulties with harder questions as easier questions are thought to be more commonly encountered during its training.

Consequently, questions that pose substantial challenges for human examinees also present difficulties for ChatGPT. Future studies should explore ChatGPT learning potential through longitudinal studies utilizing extensive question databases over a longer follow-up period.

Furthermore, an unexpected observation was made regarding the relationship between question length and ChatGPT accuracy. Longer question stems were associated with a higher likelihood of incorrect answers. Our analysis revealed a negative correlation (*r*_s_ = −0.076, *p* < 0.001), indicating that correctly answered questions had shorter average lengths (1304 ± 359 characters) compared to incorrectly answered questions (1346 ± 325 characters) with *p* = 0.001. Although longer questions may contain more information that could potentially assist ChatGPT in providing precise answers, the analysis of additional, sometimes unnecessary and distracting information may impose a higher computational load to the language-based AI model. This increased cognitive effort can lead to a greater probability of errors as ChatGPT navigates its decision-making pathway process. In simpler terms, an excess of information may result in an excess of possible pathways, increasing the chances of incorrect responses [22]. Test question writers should keep this trend in mind by including additional information not essential to answer the question to increase (processing) complexity, rather than just making questions longer, to make them more “AI-proof.”

Upon analyzing the questions answered incorrectly by ChatGPT, we made an intriguing observation. In many instances, ChatGPT selected answers that were chosen by less than 5% or sometimes even 0% of the AMBOSS© peer group users. When answering test questions correctly, the correct answer choice was also selected by 68.2 ± 16.7% of the human AMBOSS© peer group users. However, when answering incorrectly, ChatGPT frequently chose answers that had been selected by only 16.1 ± 12.3% of the human AMBOSS© peer group users. In these cases, the correct answer was however selected by the majority of the human AMBOSS© peer group users with 57.2 ± 17.7%. This suggests that ChatGPT occasionally chose completely incorrect answers instead of the second-best option, which was selected more frequently by the peer group. This finding potentially highlights once again the decision-making pathway process of ChatGPT with its tendency to selectively incorporate specific information from the question text into its analysis, while omitting other crucial details necessary for accurate responses. This observation effectively shows how ChatGPT is capable of opting in answer options but does poorly at opting out answer options.

As AI continues to be integrated in medical education, ChatGPT presents a valuable tool for students preparing for exams like the USMLE. Albeit current limitations, such as difficulties with complex question formats, suggest areas for further refinement, students can already effectively integrate ChatGPT for active learning. It can aid in reviewing clinical reasoning principles, practicing standard question types, and simulating exam scenarios. Additionally, ChatGPT delivers detailed explanations in seconds, allowing students to quickly access background information without the need to consult multiple sources, making it easier to address knowledge gaps. By prompting ChatGPT to explain concepts in different ways, students can receive personalized explanations tailored to their individual learning needs. When used alongside traditional study materials, ChatGPT can serve as a powerful complementary resource to optimize exam preparation and deepen understanding.

A major strength of our study was the utilization of an extensive practice question bank for the USMLE® Step 2CK, kindly granted permission by AMBOSS©. This enabled us to categorize questions based on various medical categories, question types, and difficulties, facilitating an in-depth and insightful analysis of ChatGPT computational capabilities. These findings have important implications for the field of medical education and can contribute to further improvements in the training and development of AI systems in healthcare.

Stemming from our analysis of 3000 ChatGPT-answered USMLE® Step 2CK questions, also test question writers may consider the following evidence-based recommendations to render their exams ChatGPT-proof.

(i) We found that ChatGPT scored significantly lower (27.1% vs. 58.2%; *p *<0.001) when answering table-based questions compared to standard questions. Table-based questions assess the candidate’s profound understanding of physiological relationships in the human body by analyzing the anticipated changes of different parameters in a myriad of clinical scenarios (e.g., medical condition, start of medical treatment). These questions aim to assess the candidate’s ability to evaluate the patient’s condition and guide clinical decision-making. Therefore, this question type requires an increased level of integrative thinking via the inclusion and exclusion of answer choices based on each parameter. This is a test-taking skill ChatGPT barely possesses in its current state. Therefore, to make test questions more ChatGPT-proof, increasing the proportion of table-based questions out of all test questions is advisable.
(ii) We calculated that the mean number of characters was 1304 ± 359 vs. 1346 ± 325 for correct and incorrect answers provided by ChatGPT, respectively with *p *= 0.001. To make USMLE® Step 2CK practice questions ChatGPT-proof, making longer question stems by including more—and also non-necessary—information is advisable.
(iii) The inability of ChatGPT 3.5 to analyze images could have been effectively used to create more AI-resistant questions by incorporating media such as images, rather than relying solely on text. However, with newer versions now capable of image analysis, this possibility must be reconsidered, as future versions of AI will likely peu á peu overcome this limitation.
(iv) Our analysis revealed that specific categories such as *Immune System* and *Multisystem Processes & Disorders* were particularly challenging for the chatbot. Interestingly, these question categories were characterized by a greater percentage of table-based questions: overall, table-based questions comprised 1.6% of all questions while in both categories they comprised 4.5% (3 out of 67 questions) and 2.7% (6 out of 217 questions), respectively. Thus, the remaining specialties may adapt their test question set toward a more table-based test question style. Yet, despite the higher proportion of table-based questions, these categories showed an overall lower performance also in the AMBOSS© user peer group with 55.7% for *Immune System* and 61.6% for *Multisystem Processes & Disorders* when compared to the overall average of 63.6% indicating a greater level of difficulty for these categories. This is also being reflected in the level of difficulty as given by AMBOSS©: The average number of hammers was 2.79 ± 1.1 for *Immune System* and 2.50 ± 1.2 for *Multisystem Processes and Disorders* when compared to the overall average of 2.36 ± 1.1.

This study, however, is not free of limitations. Firstly, future studies employing newer versions of ChatGPT, such as ChatGPT 4, will need to investigate whether the performance shown in this study holds true in the context of USMLE Step 2CK–like questions, particularly in assessing procedural knowledge in varied clinical scenarios. Establishing a robust benchmark with ChatGPT 3.5, as done in our study, sets a foundational comparison for evaluating the enhanced computational capabilities of subsequent models. Secondly, the uneven distribution of question categories, such as the overrepresentation of questions pertinent to the nervous system, limits the generalizability and comparability of our findings. Although this reflects the natural composition of the question bank, it might introduce bias that may ultimately affect conclusions about ChatGPT’s performance in certain specialties. Future studies should include larger numbers of questions from the same categories to validate or challenge the findings presented here. Thirdly, albeit our study revealing interesting statistically significant relationships, this does not inherently imply correlation or causation. Further research in the complex and continuously developing intersection of AI and medical education is required to our findings and better understand the complex relationship between question characteristics and ChatGPT’s performance.

In summary, we herein present simple, yet robust, safety measurements to counteract the risk of ChatGPT-based test cheating. This study represents the to-date largest and most comprehensive effort to analyze the performance of ChatGPT on USMLE® Step 2 CK questions. Hence, ChatGPT can be considered a curse and a blessing for medical education. While ChatGPT excels at condensing medical knowledge and integrating clinical reasoning principles in a widely accessible and easy-to-use interface, ChatGPT also poses the risk of developing a tunnel view of medical data and clinical knowledge by oversimplifying the reasoning required to arrive at a diagnosis. Further, ChatGPT might be misunderstood as a tempting tool to cheat rather than study for career-deciding exams such as the USMLE® Step 2CK. However, our atlas of ChatGPT performance on 3000 USMLE® Step 2 CK paints a more realistic picture of this chatbot with its untapped potential still surpassing its actual potency. Overall, we call on medical education stakeholders to integrate ChatGPT as soon as possible into the medical curriculum, generate ChatGPT-proof test questions, and exploit this revolutionary technology for the good of medical students.

## Conclusion

This study aimed to evaluate the performance of ChatGPT, a language-based AI model powered by machine learning mechanisms and deep learning strategies. The results provided a comprehensive understanding of ChatGPT proficiency in answering the to-date largest number of the career-deciding USMLE® Step 2 CK questions: Overall, ChatGPT was able to achieve an accuracy rate of 57.7%, falling slightly below the passing threshold of approximately 60%. Yet, compared with the AMBOSS© user peer group, ChatGPT was able to score at the 53^rd^ percentile, indicating an average test-taking performance. Differences in performance were found when analyzing the questions based on category, question type, and difficulty enabling test question writers to adapt question style to make practice and exam questions ChatGPT-proof. Leveraging our findings, medical educators can enhance the integrity of their assessments while making informed decisions about the integration of AI tools such as ChatGPT into their teaching strategies. For example, educators can increase the proportion of table-based questions—where ChatGPT struggled significantly—to design more AI-resistant exams that better assess deep clinical understanding. For students, recognizing the model’s limitations allows them to use ChatGPT as a supplemental resource rather than a study replacement. For instance, students can employ ChatGPT to support their learning in straightforward case-based scenarios, while relying on traditional methods to successfully answer more complex physiological relationships. This study contributes to our understanding of AI applications in medical education, specifically ChatGPT performance in answering medical licensing examination questions. Future research should focus on refining AI models for improved accuracy, including addressing limitations in comprehending table-based questions. Ultimately, AI-powered systems like ChatGPT have the potential to advance medical education and enhance clinical decision-making, but further investigations and refinements are needed to optimize their contribution to healthcare practices.

## Data Availability

All data generated or analyzed during this study are included in this published article and its supplementary information files.
